# Shambhala: a platform-agnostic data harmonizer for gene expression data

**DOI:** 10.1186/s12859-019-2641-8

**Published:** 2019-02-06

**Authors:** Nicolas Borisov, Irina Shabalina, Victor Tkachev, Maxim Sorokin, Andrew Garazha, Andrey Pulin, Ilya I. Eremin, Anton Buzdin

**Affiliations:** 1 I.M. Sechenov First Moscow State Medical University, Sechenov University, Moscow, 119991 Russia; 2 Department of bioinformatics and molecular networks, OmicsWay Corporation, Walnut, CA USA; 3 Faculty of Mathematics and Information Technologies, Petrozavodsk State University, Anokhina str., 20, Petrozavodsk, 185910 Russia; 4 Group for Genomic Regulation of Cell Signaling Systems, Shemyakin-Ovchinnikov Institute of Bioorganic Chemistry, Moscow, 117997 Russia; 5 Laboratory of Bioinformatics, Oncology and Immunology, D. Rogachyov Federal Research Center of Pediatric Hematology, Moscow, 117198 Russia; 6 Laboratory for Cell Biology and Developmental Pathology, Federal State Institution “Institute of General Pathology and Pathophysiology”, FSBSI “IGPP”, Moscow, Russia; 7 Department for Regenerative Medicine, JSC Generium, Moscow, Russia

**Keywords:** Transcriptome, Gene expression, Microarray hybridization, Next-generation sequencing, Harmonization of transcriptional profiles, Comparison of multiple datasets

## Abstract

**Background:**

Harmonization techniques make different gene expression profiles and their sets compatible and ready for comparisons. Here we present a new bioinformatic tool termed Shambhala for harmonization of multiple human gene expression datasets obtained using different experimental methods and platforms of microarray hybridization and RNA sequencing.

**Results:**

Unlike previously published methods enabling good quality data harmonization for only two datasets, Shambhala allows conversion of multiple datasets into the universal form suitable for further comparisons. Shambhala harmonization is based on the calibration of gene expression profiles using the auxiliary standardization dataset. Each profile is transformed to make it similar to the output of microarray hybridization platform Affymetrix Human Gene. This platform was chosen because it has the biggest number of human gene expression profiles deposited in public databases. We evaluated Shambhala ability to retain biologically important features after harmonization. The same four biological samples taken in multiple replicates were profiled independently using three and four different experimental platforms, respectively, then Shambhala-harmonized and investigated by hierarchical clustering.

**Conclusion:**

Our results showed that unlike other frequently used methods: quantile normalization and DESeq/DESeq2 normalization, Shambhala harmonization was the only method supporting sample-specific and platform-independent biologically meaningful clustering for the data obtained from multiple experimental platforms.

**Electronic supplementary material:**

The online version of this article (10.1186/s12859-019-2641-8) contains supplementary material, which is available to authorized users.

## Background

Public repositories of gene expression data cover a rich spectrum of normal and pathological conditions, including all known human diseases and developmental features [1–4]. The most popular repositories such as Gene Expression Omnibus (GEO) [3] and Array-Express [4] accumulate data for more than 2 million of individual expression profiles in more than 70,000 series of experiments. These transcriptional profiles were generally obtained using different experimental modifications of microarray hybridization and RNA sequencing. However, the expression data is poorly comparable among the different experimental datasets [5–9]. This problem is due to both (i) technical features linked with the experimental platforms, and (ii) so-called batch effect [10]. The latter term means that even the experimental results obtained using the same reagents and on the same equipment can be significantly biased over time.

This non-comparability of gene expression data hampers further levels of data analysis for the different datasets, e.g. finding differentially expressed genes and assessing activation of molecular pathways [11, 12].

To solve this problem, the data must be either normalized (when datasets under comparison were obtained using one experimental platform) or harmonized (when different platforms were used) [12]. For the normalization, more attention is paid to mere equilibration of the scaling factors. Contrarily, for most cases of the harmonization, there is a need to reshape distributions for the entire gene expression profiles.

The normalization methods include quantile normalization (QN) [13], frozen robust multi-array analysis for microarray hybridization data (FRMA) [14], Empirical Bayes (EB) method also known as ComBat [15], or Differential Expression analysis for Sequence count data, DESeq [16]/DESeq2 [17]. The methods for harmonization include distance-weighted discrimination (DWD) [18, 19], cross-platform normalization (XPN) [20, 21], Quantile Discretization (QD) [22], Normalized Discretization (NorDi) [22], DisTran (Distribution Transformation) [23], Gene Quantiles (GQ) [24], and platform-independent latent Dirichlet allocation (PLIDA) [25]. In a fundamental survey of different harmonization techniques [20] the XPN method showed the best performance. The harmonization acts by deeply restructuring distributions of gene expression levels for the samples under comparison. As a rule, harmonization algorithms use data clustering to identify similarities between the gene expression profiles obtained using different experimental platforms, and then increase these similarity regions during subsequent reshaping of the expression profiles.

However, to our knowledge all previously published harmonization methods have a substantial limitation that they are capable of performing harmonization for only two expression datasets [20]. Thus, only the data from two experimental platforms can be simultaneously harmonized. Moreover, the resulting hybrid data are not further compatible with any of the existing formats for the experimental platforms. Moreover, the published methods show good performance only for the datasets of a comparable sample size, therefore complicating harmonization of the existing data.

Here, we present a new method for cross-platform data harmonization termed *Shambhala* that may be considered a more universal tool compared to the existing approaches. Unlike previous harmonizers, Shambhala is independent on (i) number of harmonized datasets and/or experimental platforms, and (ii) number of samples in every dataset. The Shambhala harmonization protocol includes several specific features such as the *auxiliary calibration dataset* that helps to initially transform the data, and the *reference definitive dataset* that defines the universal shape of the output harmonized gene expression profile. Next, we investigated the performance of Shambhala to harmonize the gene expression data from multiple experimental platforms obtained from the Microarray Quality Control (MAQC) [26] and Sequencing Quality Control (SEQC) datasets [27]. Our data evidence that being currently a unique tool for harmonization of multiple datasets, Shambhala provides outputs reflecting biological origin of a biosample rather than the experimental platform used. In contrast, other harmonization tools are not applicable to this type of tasks in principle, and the normalization tools such as QN and DESeq/DESeq2, return low-quality platform-biased outputs.

## Results

### Shambhala method rationale

We developed Shambhala method for cross-platform comparisons of multiple datasets. In its present form, the method was tailored for the comparison of human gene expression data, and its application for other organism data requires further specific data search. Let us look at the problem of cross-platform harmonization in more detail. Imagine an arbitrary set of experimental platforms that has produced a set gene expression profiles. Our goal is to make them all comparable. To do so, we may make them similar to a pre-defined reference. This reference may be taken from a set of profiles that has been obtained at a widely used experimental platform; we can term this set the *reference definitive dataset* (*Q*). The process of profile transformation involves multiple iteration steps, when the dataset *P*, which contains profiles under harmonization, is altered, whereas the dataset *Q* remains unchanged. Consequently, the output of such transform has gene expression profiles like those obtained using the same experimental platform, as for the dataset *Q.*

**Fig. 1:**
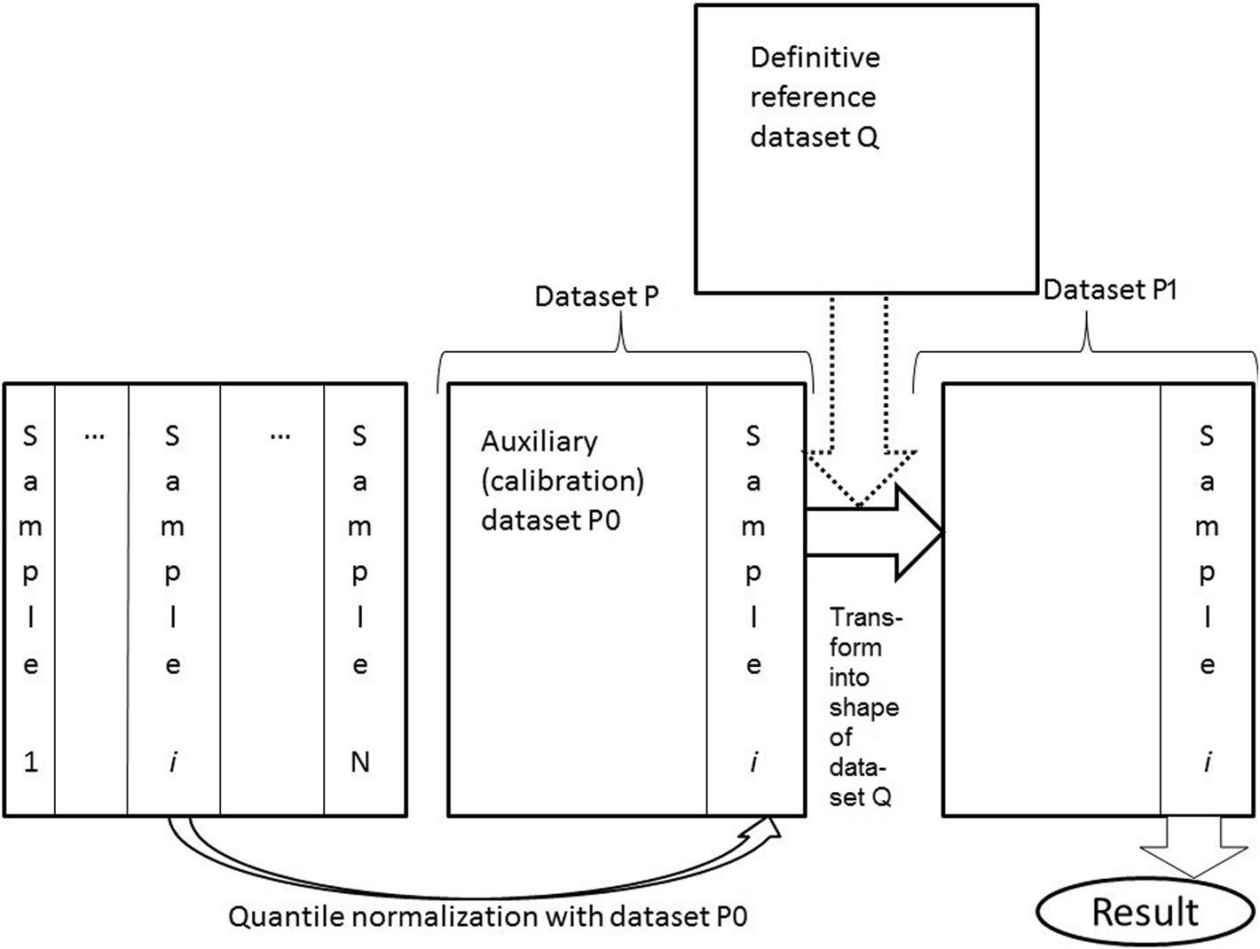
Schematic representation of Shambhala pipeline for harmonization of gene expression data. Various profiles from samples (1… *N*) obtained at different platforms are taken one-by-one, merged with an *auxiliary calibration dataset P*0 and then quantile-normalized with it. This produces the dataset *P*, which is then transformed into the shape of the definitive dataset *Q*; during transformation, only the dataset *P* changes, while *Q* remains constant. The result of such a conversion, dataset *P*1, contains the transformed profile for sample *i*, which is considered harmonized. Profiles from all other samples (1,…,*N*) are harmonized one-by-one using the same algorithm

To ensure comparable harmonization results for the datasets of different size, we developed the following procedure. The profiles from different platforms are sometimes completely different, and to make the gene expression distribution comparable for each profile before transformation into the *Q*-shape, we should equalize it using another pre-defined dataset called *auxiliary calibration dataset* (*P*0). In other words, it means that each individual gene expression profile under harmonization, say *i*, is transformed into the *Q-*shape not within the original dataset of unharmonized profiles from certain experimental platform, but rather being taken alone, and then merged with *P*0. Namely, we quantile-normalize [13] profile *i* with the dataset *P*0, which produces the dataset *P* for further transformation. This dataset *P* is then transformed into the shape of the dataset *Q*, thus producing the dataset *P*1. From this dataset *P*1, only the transformed single profile *i* is taken for further analysis. This procedure is then applied to all other gene expression profiles which need to be harmonized (Fig. 1).

Some features of this pipeline, which are used for transformation of dataset *P* into the shape of the dataset *Q*, were inspired by the XPN method [21] that showed the best performance among the pairwise cross-platform harmonization techniques [20]. Such features include stochastic clustering for gene and samples using genetic algorithms, and partially-linear iterative harmonization of two datasets. However, the major distinctions here are that (i) in the Shambhala algorithm, the dataset *P* changes, while *Q* remains constant during the iteration steps, whereas in the XPN both are transformed iteratively; (ii) to increase stability of the results, Shambhala uses spherical (cosine-based) [28, 29] rather than barycentric (as in the XPN) clustering of samples in *P* and *Q* datasets.

Importantly, the Shambhala pipeline depends on two datasets, *P*0 and *Q*, the latter acting as the reference for gene expression profiles after harmonization, and the former serving for preliminary calibration of expression level ranges. As the dataset *Q* for this application, we used the mRNA expression profiles taken from the Genotype Tissue Expression (GTEx) project [30], namely one hundred samples corresponding to ten normal human tissue types (brain, nerve, skin, adipose, muscle, heart, lung, thyroid, blood vessels and blood). Among the others, the GTEx comprised profiling using microarray platform Affymetrix Human Gene 1.1 ST (GPL16977; deposited under accession number GSE45878) and NGS platform Illumina HiSeq 2000 (accession number E-MTAB-5214). We selected the microarray GTEx results as the *Q* dataset because it is frequently considered the golden standard for microarray hybridization of human tissues [31, 32], while Affymetrix microarray-profiled expression data are the most abundant kind of data in public databases, e.g. in the Gene Expression Omnibus (GEO) database as for 2018-11-06. To investigate the influence of the definitive dataset on the performance of Shambhala harmonization, we also analyzed an alternative *Q*-set obtained using the Illumina HiSeq 2000 platform.

When selecting the optimal auxiliary calibration dataset (*P*0) for Shambhala implementation, we found that our previous experimental dataset including 39 human gene expression profiles obtained using CustomArray microchip platform (CustomArray, USA) showed the best performance in clustering tests compared to more than twenty other datasets of the comparable size (data not shown). Interestingly, our attempts to use the GTEx dataset for both *P*0 and *Q*, have failed to produce good sample clustering.

### Shambhala method validation and harmonization quality assessment

To investigate the robustness and quality of Shambhala approach, we took a model of gene expression profiles obtained for the same biosamples using different experimental platforms.

We used published gene expression data from the Microarray Quality Control [26]; GEO accession number GSE5350) and Sequencing Quality Control, SEQC [27]; GSE47792 and GSE56457) projects (Table 1). Both MAQC and SEQC projects investigated compatibilities of gene expression profiles obtained using various microarray and sequencing platforms for the same set of four sample types (named A, B, C, D), each done in multiple replicates. Type A samples were the commercially available Stratagene Universal Human Reference RNA specimens for all but brain human tissues; type B samples – also commercially available the Ambion Human Brain Reference RNA. Type C and D samples were the mixtures of A and B with the A:B ratios of 3:1 and 1:3, respectively. Type C sample, therefore, was biologically closer to the sample A, and type D – to the sample B.

**Table 1:** MAQC and SEQC project data used for Shambhala validation

Project	GEO reference	Platform name	Platform GEO ID	Number of samples
MAQC	GSE5350	Agilent-012391 Whole Human Genome Oligo Microarray G4112A ()	GPL1708	59
MAQC	GSE5350	Affymetrix Human Genome U133 Plus 2.0 Array	GPL570	59
MAQC	GSE5350	Illumina Sentrix Human-6 Expression Beadchip	GPL2507	59
SEQC	GSE47792	Illumina HiSeq 2000	GPL11154	1324
SEQC	GSE56457	Illumina HumanHT-12 V4.0 expression beadchip	GPL10558	24
SEQC	GSE56457	Affymetrix Human Gene 2.0 ST Array	GPL17930	16
SEQC	GSE56457	Affymetrix GeneChip® PrimeView™ Human Gene Expression Array	GPL16043	16

The MAQC project investigated the expression profiles for 14–15 technical replicates of all sample types, A to D, for the most popular microarray platforms, including Agilent-012391 Whole Human Genome Oligo Microarray G4112A (GPL1708), Affymetrix Human Genome U133 Plus 2.0 Array (GPL570) and Illumina Sentrix Human-6 Expression Beadchip (GPL2507). In the SEQC project, the microarray expression profiles for the same biosamples were compared with the RNA sequencing data obtained using Illumina HiSeq 2000 platform (GPL11154), see Table 1.

To assess quality of data harmonization, we tested whether hierarchical clustering of the harmonized genes expression profiles will be biologically meaningful or rather dependent on the experimental platforms used. For the clustering, Euclidean distance was used as a metric of proximity. An ideal method for data harmonization would allow grouping of output expression profiles according to the type of biosamples (A to D), but not according to a platform used. Similar types of biosamples (type A and C, and type B and D) were expected to show more tight clustering. In contrast, the platform-based clustering independent on the biological similarities of biosamples could be considered bad result.

**Fig. 2:**
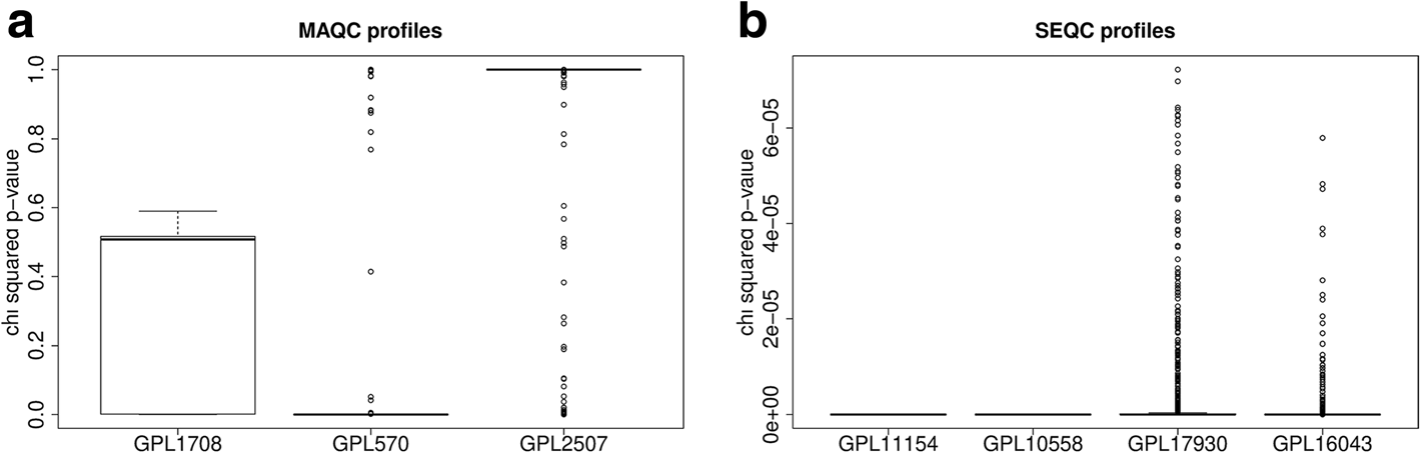
Pearson chi squared test *p*-value for gene expression levels. The null hypothesis was that gene expression level do not match the negative binomial law. The optimal parameters for negative binomial distribution for every gene were first assessed using the *glm.nb* R function, and then the applicability of negative binomial law was checked using the *chisq.test* function. Panel **a**: MAQC data (platforms Agilent GPL1708, Affymetrix GPL570, Illumina GPL3507). Panel **b**: SEQC data (platforms Illumina HiSeq 2000 GPL11154, microarray platforms Illumina GPL10558, Affymetrix GPL17930 and GPL16043)

To test Shambhala, we took data from three experimental platforms for MAQC dataset and from four platforms for SEQC. All gene expression profiles were harmonized using three alternative methods:

1. Quantile normalization, QN [13].
2. Differential expression analysis for sequence count data, DESeq [16]/DESeq2 [17] using the *estimateSizeFactors* module. To make the microarray data formally suitable for DESeq/DESeq2 normalization, we took an integer part of all microarray-measured expression level values for each gene and each sample. The intensity values for microarray-measured signal were taken as they were deposited in GEO repository, i.e. after device-dependent primary background correction or equilibration but before any cross-platform transformation or harmonization. Although the DESeq/DESeq2 method was designed for normalization of NGS data and assumes that the count data follow a negative binomial distribution, there were several examples when DESeq/DESeq2 was formally applied to rounded microarray data, both in model investigations based on microarray profiles [34] and for processing human patient’s data [35, 36]. Moreover, having applied the negative binomial regression followed by the Pearson chi-squared test, we found that although the MAQC microarray gene expression values were not distributed according to negative binomial law (particularly for the Illumina GPL2507 and Agilent GPL1708 platforms; Fig. 2a), the SEQC microarray profiles (platforms Illumina GPL10558, as well as Affymetrix GPL17930 and GPL16043) matched the negative binomial distribution (Fig. 2b).
3. Shambhala harmonization with two different GTEx definitive datasets (obtained using either microarray Affymetrix or NGS Illumina HiSeq 2000 platforms). Shambhala method was compared with other above normalization techniques (QN, DESeq/DESeq2) because they are popular tools used for merging data from multiple datasets. The standard harmonization methods such as XPN [20, 21] are not applicable because they enable comparisons of only up to two datasets.

### Performance test for three-platform data harmonization

We tested Shambhala, QN and DESeq/DESeq2 methods for their abilities to simultaneously harmonize data from three experimental microarray platforms (Affymetrix GPL570, Agilent GPL1708 and Illumina GPL2507) from the MAQC project.

**Fig. 3:**
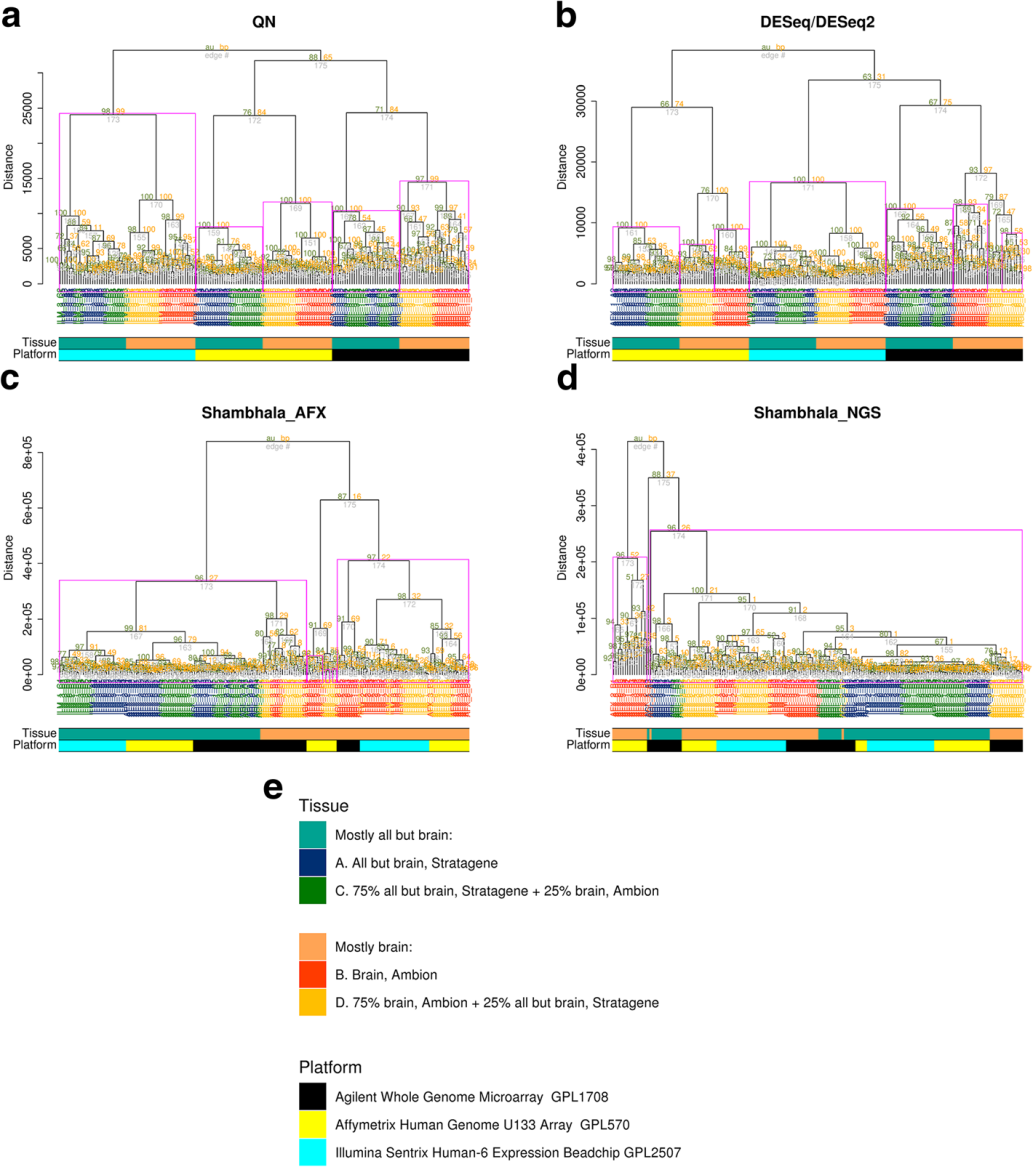
Hierarchical clustering at the level of individual gene expression for MAQC project data. Panel **a** – results following quantile normalization (QN); **b** – DESeq/DESeq2; **c** – Shambhala with Affymetrix microarray *Q*-dataset; **d** – Shambhala with Illumina HiSeq 2000 *Q*-dataset. Panel **e** – legend explaining origin of biosamples A, B, C, D and experimental platform in the project. More detailed view of the dendrograms is given in Additional file 5

The results (Fig. 3) suggest that the clustering following QN (Fig. 3a) and DESeq/DESeq2 (Fig. 3b) both occur on a platform-specific basis that ignores the biological nature of biosamples under comparison. All the expression profiles are clustered into the three major groups specific only to the microarray platforms used (shown by cyan, yellow and black markers on the figure). In contrast, following Shambhala harmonization with Affymetrix definitive dataset (Fig. 3c) we observed sample type-specific clustering where the biologically similar samples A + C and B + D formed clear-cut separate clusters. Shambhala harmonization with HiSeq 2000 definitive dataset produced results of an intermediate quality between Shambhala with Afftmetrix *Q*-set and QN/DESeq2 normalization (Fig. 3d). However, neither algorithm could correctly distinguish between the samples A and C or B and D, which is the obvious limitation of our approach.

### Performance test for four-platform data harmonization

We next compared the abilities of Shambhala, QN and DESeq/DESeq2 methods to harmonize the data obtained using four experimental platforms. To this end we took the gene expression profiles from the Sequencing Quality Control (SEQC) project [33], Table 1. In this case, we harmonized data obtained for three microarray platforms and one RNA sequencing platforms, Illumina HumanHT-12 V4.0 (GPL10558), Affymetrix Human Gene 2.0 ST (GPL17930), Affymetrix GeneChip PrimeView (GPL16043), and Illumina HiSeq 2000 (GPL11154), respectively. For RNA sequencing data, we applied filtering to remove profiles with low, and, therefore, unreliably measured, numbers of mapped reads (Additional file 1). Following filtering, we identified for further comparisons 5486 reliable genes out of the initial set of 17,567 genes.

**Fig. 4:**
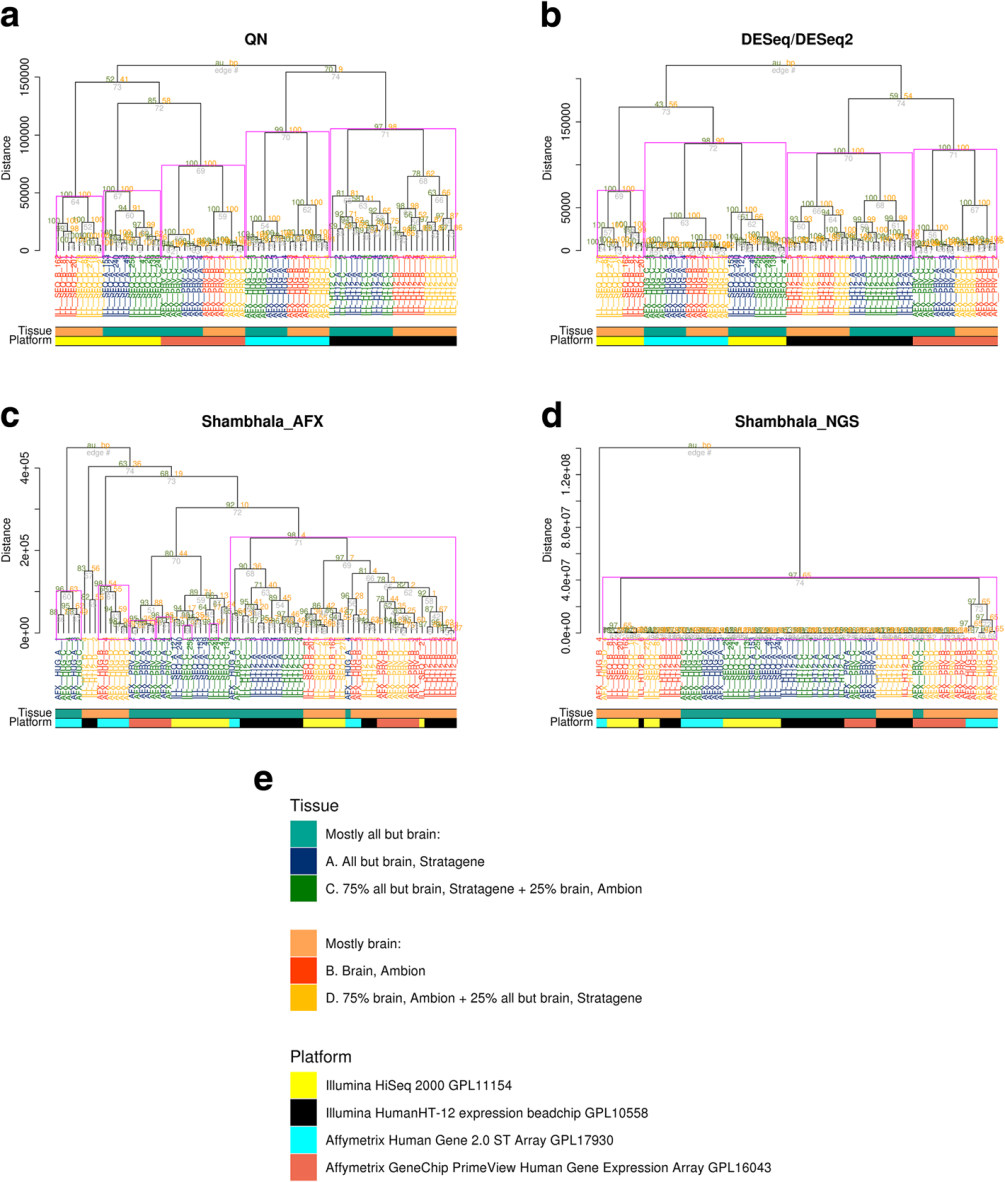
Hierarchical clustering at the level of individual gene expression for SEQC project data. Panel **a** – results following quantile normalization (QN); **b** – DESeq/DESeq2; **c** – Shambhala with Affymetrix microarray *Q*-dataset; **d** – Shambhala with Illumina HiSeq 2000 *Q*-dataset. Panel **e** – legend explaining origin of biosamples A, B, C, D and experimental platform in the project. To facilitate the visual analysis of the hierarchical clustering dendrogram, we selected randomly only 20 profiles out of 1324 that were obtained using the Illumina HiSeq 2000 (GPL11154) platform. More detailed view of the dendrograms is given in Additional file 6

The results obtained (Fig. 4) suggest that as in the previous case, the QN and DESeq/DESeq2 methods provide purely platform-specific outputs ignoring the biological composition of biosamples tested (Fig. 4a and b, respectively; platforms indicated by the lower marker), thus giving four major clusters specific to the above experimental platforms.

However, the Shambhala algorithm outputs with microarray Affymetrix *Q*-dataset (Fig. 4c) again supported biological type-specific clustering for most of the samples, irrespective of their experimental microarray or sequencing platform. Again, the performance of Shambhala with Illumina HiSeq 2000 *Q*-dataset (Fig. 4d) was better than QN and DESeq/DESeq2 but worse than for the Affymetrix *Q*-dataset. To our knowledge, this was the first study when the microarray and RNA sequencing data were successfully harmonized. However, as before, the biologically similar A + C and B + D sample types were merged on the dendrogram, which most probably stresses natural limitations of the Shambhala harmonization tool (Fig. 4c).

**Fig. 5:**
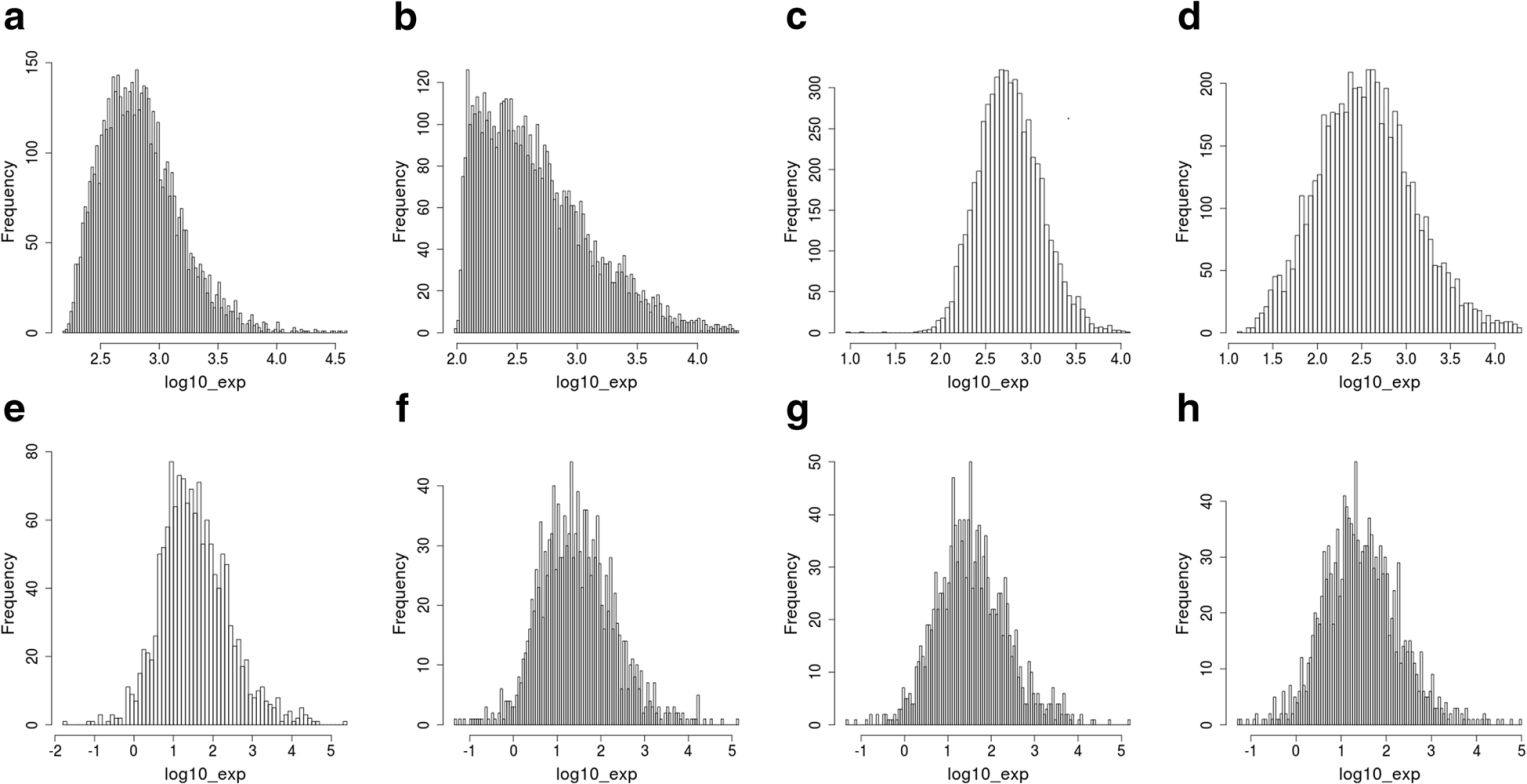
Averaged expression profile for samples of type A before (upper row, panels **a** to **d**) and after (lower row, panels **e** to **h**) the Shambhala harmonization. The profiles were obtained using the platforms Illumina HiSeq 2000, GPL11154 (panels **a** and **e**), Illumina HumanHT-12 V4.0 expression beadchip, GPL10558 (**b** and **f**), Affymetrix Human Gene 2.0 ST Array, GPL17930 (**c** and **g**), and Affymetrix GeneChip PrimeView Human Gene Expression Array, GPL16043 (**d** and **h**)

In should be mentioned that for all the platforms investigated, Shambhala tool produced uniformly shaped and similarly distributed gene expression density profiles (Fig. 5), thus confirming its ability to standardize various types of experimental outputs; note the initial distribution profiles were highly different among the experimental platforms.

## Discussion

Although attempts to develop universal cross-platform transcriptome harmonization technique are known for more than a decade, the acceptable performance was shown before only for harmonization of up to two expression datasets [20, 21]. In this study, we developed a new method termed Shambhala suitable for the universal, platform-agnostic harmonization. Unlike previous techniques, Shambhala enables simultaneous harmonization of multiple gene expression datasets, with the standardized uniformly shaped gene expression output. We used the rationale of transforming the experimental expression profiles into the shape of a pre-selected known gene expression platform. Transformation of different sample profiles into the standard definitive form is done for all profiles independently upon other profiles under harmonization. Another distinguishing feature of Shambhala protocol is that any single profile cannot be transformed alone into the definitive shape. Instead, it should be reshaped into to the *Q*-form within an auxiliary calibration dataset (*P*0-dataset).

In this study, we tried two sets of expression profiles (obtained using microarray Affymetrix and Illumina HiSeq 2000 platforms) from the GTEx project [30] as the reference definitive dataset, and the MAQC [26] and SEQC [27] datasets for validation of Shambhala algorithm. The latter two datasets were selected because they contain gene expression data for the same four types of biosamples profiled using different experimental platforms.

The criteria for selecting the auxiliary calibration dataset (*P*0-dataset) were to provide the best merging of biologically relevant profiles after harmonization. During the training stage, we selected the *P*0-dataset, which could ensure the good-quality harmonization of the MAQC dataset, namely for the profiles obtained using the Affymetrix and Agilent microarray platforms. Importantly, we did not observe good clustering quality when trying the same GTEx dataset as both *P*0 and *Q*, so we had to select another dataset (originated from the CustomArray platfrom) as *P*0.

We validated Shambhala performance for three experimental platforms from the MAQC and four – from the SEQC dataset. In the latter case, three microarray platforms were merged with one RNA sequencing platform. Shambhala could effectively convert the transcriptomes from multiple platforms, into a standard uniformly shaped form (Fig. 5). In both cases, we showed that Shambhala method significantly outperformed the existing agnostic multi-platform normalization tools, QN [13] and DESeq/DESeq2 [16, 17]. Unlike the other methods, Shambhala could allocate biological sample type-specific clustering of the expression profiles, even for the comparison of microarray versus RNA sequencing data. The highly similar biosamples A and C could be efficiently distinguished from biosamples B and D, also highly similar. Type C and D samples were the mixtures of A and B. Type A, therefore, was 100% A, type B – 100% B, type C – 75% A and 25% B, type D – 25% A and 75% B. However, in neither trial could the algorithm distinguish between the A vs C, or B vs D biosamples. Nevertheless, the method may afford simultaneous harmonization of any number of transcriptomes obtained using any number of experimental platforms; the method’s quantitative performance is only limited by the capacity of a hardware used and/or calculation facilities.

The Shambhala performance with the NGS reference definitive dataset appeared better than for QN or DESeq2 normalization, but somewhat worse than for Shambhala with microarray Affymetrix reference dataset.

In the present form Shambhala data harmonizer tool was implemented only for the human gene expression data, with the species-specificity being dependent on the reference definitive and auxiliary calibration datasets. Its further adaptation to other organisms is a technical task that would require a representative sampling of gene expression data to complete good quality *P*0 and *Q* datasets.

Finally, we suggest that the Shambhala approach, or its further modifications, can be a perspective candidate for a massive platform-agnostic harmonization technique enabling direct comparisons of the data accumulated in different laboratories using different equipment and reagents.

## Conclusion

We presented here a new approach, termed Shambhala, to universal harmonization of gene expression profiles obtained using multiple experimental platforms, for both microarray hybridization and RNA sequencing methods. In this application, Shambhala algorithm was tuned and applied for the comparisons of human gene expression profiles. During harmonization, every single gene expression profile is transformed into the definitive shape using the reference gene expression dataset. We showed that unlike any previous methods, Shambhala may enable biologically meaningful harmonization of gene expression data obtained using three or four experimental platforms.

## Methods

### Shambhala harmonizer implementation

The code for Shambhala was written as further modification and upgrade of the R package CONOR [20]. The whole code was arranged as the R package HARMONY. This package, as well as a code example for Shambhala application are deposited at Github, https://github.com/oncobox-admin/harmony.

The cluster dendrograms were built using R package *dendextend*. The reliability of hierarchical clustering was assessed with the bootstrap procedure using the R package *pvclust*.

## Additional files

Additional file 1: Description and validation of the reliability filter for the results of NGS gene expression profiling (DOCX 204 kb)

Additional file 2: Definitive (*Q*) and auxiliary calibration (*P*0) datasets for the Shambhala method. (XLSX 42198 kb)

Additional file 3: Harmonized MAQC gene expression profiles. (XLSX 45943 kb)

Additional file 4: Harmonized SEQC gene expression profiles. (XLSX 219245 kb)

Additional file 5: A detailed view of hierarchical clustering for gene expression levels for MAQC project data after application different harmonization methods. (PPTX 857 kb)

Additional file 6: A detailed view of hierarchical clustering for gene expression levels for SEQC project data after application different harmonization methods. (PPTX 647 kb)

## Abbreviations

DESeq: Differential expression of RNA-seq data
DWD: Distance-weighted discrimination
EB: Empirical Bayes
FRMA: Frozen robust multi-array analysis
GEO: Gene expression omnibus
GQ: Gene quantiles
GTEx: Genotype-tissue expression
MAQC: Microarray quality control
PLIDA: Platform-independent latent Dirichlet allocation
QD: Quantile discretization
QN: Quantile normalization
SEQC: Sequencing quality control
XPN: Cross-planform normalization
