# Supplementary material for: Shambhala: a platform-agnostic data harmonizer for gene expression data
Source: BMC Bioinformatics. 2019 Feb 6;20:66. doi: 10.1186/s12859-019-2641-8 (PMC6366102; doi:10.1186/s12859-019-2641-8)
Supplement: Supplementary file 1 — Description and validation of the reliability filter for the results of NGS gene expression profiling (DOCX 204 kb) [file 12859_2019_2641_MOESM1_ESM.docx]

**Supplementary material 1: Filter for the results of NGS gene expression profiling**

***Background: filtering genes with low numbers of aligned reads***

It seems obvious that the accuracy of measuring low-abundant transcripts significantly depends on the sequencing depth. To improve reliability of NGS results, we applied filtering of genes with insufficiently low numbers of reads before harmonization procedures. The rationale for design of a filter is as follows. At the first iteration, we admit that the chance to register an individual type of mRNA molecule does not depend on its length. Let also *N* be the number of copies for a given type of mRNA molecules in the cell. Considering the number of protein-coding genes is ~20 000, the cell contains 2·10^4^·*µ*(*N*) copies of different genes, where *µ*(*N*) is the mean number of mRNA copies over the whole ensemble of protein-coding genes. Under such circumstances, the probability to register in one (elementary) gene reading act a given type of transcripts, which exists in a cell in *N* copies, equals to$p_{0}=\frac{N}{2\cdot10^{4}\cdot\mu(N)}$.

Assuming there are *K* total aligned NGS reads, the overall probability (*P) not* to encounter a given type of gene transcript, which is presented in the sample by *N* copies is expressed by the formula:$P=\left(1-p_{0}\right)^{K}.$Thus, taking into account that the value of *p*_0_ is extremely low compared to 1, the required number of mapped sequencing reads sufficient for registration of this transcript with the probability (1 – *P*) will be as follows: $K=\frac{\log P}{\log\left(1-p_{0}\right)}\approx\frac{-\log P\cdot2\cdot10^{4}\cdot\mu(N)}{N}.$ (1)

For the biological experiments, *P* is often set to 0.05, which corresponds to registering *at least one transcript* with 95% probability that is presented by *N* copies in *K* mapped sequencing reads.

However, most of commercially available mRNA NGS platforms generate sequencing reads which are than whole gene transcripts. In this case, the number of fragments that may present in the mixture for a transcript with the length *L* nucleotides, presented by *N* copies, is $\nu=N\cdot L$ .

The probability of registering a fragment of this gene in one reading act, therefore, equals to$\pi_{0}=\frac{\nu}{\sum_{i=1}^{2\cdot10^{4}} v_{i}}$, where the index *i* runs through all unique types of transcripts in the cell. Consequently, the number of mapped sequencing reads required to find at least one fragment of a gene with the length *L* and expression level of *N* copies per sample is $\hat{K}=\text{log}_{1-\pi_{0}}P=\frac{\log P}{1-\pi_{0}}\approx\frac{-\log P\cdot\sum_{i=1}^{2\cdot{10}^{4}} N_{i}\cdot L_{i}}{N\cdot L}$ .(2)

The major problem, nevertheless, is related to assessment of typical distribution of *N* over all the specific transcript types in the sample. Although the range of expression levels for common high-throughput mRNA profiling techniques, both microarray hybridization [1–4] and NGS [1, 5–7] does not exceed four-five orders of magnitude, the results of quantitative RT-PCR examination suggest wider span of expression levels – up to seven orders of magnitude [8, 9]. Moreover, quantitative RT-PCR is thought to measure gene expression more accurately compared to the other abovementioned methods [10].

To adapt the SEQC [11] span of expression levels to those observed in quantitative RT-RCR measurements, we did a log-linear rescaling for the expression data obtained from the SEQC project: log_10_ *N_true_* = *a*·log_10_ *N*_SEQC_ + *b*, where *a* and *b* parameters assure that the minimal and maximal true expression levels in the whole gene set are equal to 1 and 10^7^, respectively.

The dependence of required read depth (*K*) upon the number of gene copies (*N*) may be plotted as follows (see Figure S2_1, panel **a**). The blue line represents the trivial trend without correction on the gene length (Eq. (1)). The green cloud shows results that do not include mRNA length correction (Eq. (2)). The median transcript length, according to the New Human Genome Reference Sequence (GRCh37) database [12], equals to 766 nucleotides, whereas the minimal is 24, and maximal is 21246. The red dots mark the 95% quantile for the distribution of the value *K* at a fixed *N*; this dependence shows what should be the sequencing depth to find at least 95% individual types of mRNA with the probability of 95% for each of these types.

Since the true number of mRNA copies *N* is unobservable and not measureable, in order to determine the genes, whose expression levels are measured reliably, we have to exclude this explicitly formulated value from our expression filter. The panel **b** on Figure S2_1 shows the probability density (*f*), as well the complementary cumulative distribution function (survival function) for the number of gene copies *N* in the whole gene set. This survived fraction (1-*F*) of genes with reliably high number of counts (which should survive after filtration), as a function of read depth (*K*), is shown on panel **c** of Figure S2_1.


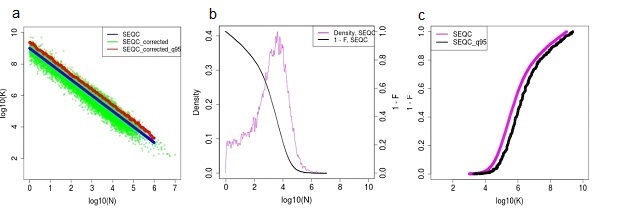


Figure S2_1 – Calibration curves for the threshold expression filter depth. Threshold shows sequencing depth sufficient for finding at least 95% of individual types of mRNA with the probability of 95% for each of these types.

Panel **a**: logarithmic scale for number of sequencing reads (*K*) sufficient to identify a type of mRNA presented by *N* copies at 95% probability. Gene expression levels were taken from the GTEx reference dataset [1]. Blue curve: assessment adapted for the range of expression levels from 1 to 10^7^ copies per cell [8–10]. Green dots: values adjusted by the factor of length for each mRNA molecule according to the GRCh38 human gene reference data [13]. Red dots: 95% quantile for the values defined above as green dots.

Panel **b**: probability density function (magenta curve) and complementary cumulative distribution function (survival function 1-*F*; black curve) for the relative distribution of mRNA copy numbers. The value of (1-*F*) equals to fraction of mRNA types with sufficient high copy numbers to survive filtering.

Panel **c**: 1-*F*, fraction of mRNA types having sufficient numbers of reads to survive filtering, as a function of sequencing depth, *K*. Magenta dots: distribution of gene expression levels from SEQC project [11] adapted for the default span of expression levels from 1 to 10^7^ [8–10]. Black dots: fraction of unique genes taken from the SEQC reference dataset that survived filtering assuring the 95% probability for registering of at least one copy of 95% mRNA types for protein coding genes. Magenta dots: distribution of gene expression levels from SEQC project adapted for the default span of expression levels from 1 to 10^7^ cell [8–10].

***Expression filter uncovers pattern of miRNA influence on translation in differentiation of myotubes.***

In a recent study, de Klerk and coauthors report results of multi-omics profiling (mRNA sequencing, ribosome-associated microRNA sequencing (ribo-seq) and microRNA sequencing), which may highlight the molecular mechanisms of gene expression regulation at the level of translation, especially its regulation by microRNAs [14] (European Nucleotide Archive, ENA, accession number PRJEB7207). The authors studied mouse myoblasts, which were specifically differentiated into myotubes. We applied the expression filter to the above sequencing data (Table S2_1). For the detailed analysis, we took the cyclohexamide-assisted data for the ribo-seq, because this type of data was reported to be less vulnerable to systemic bias in comparison with the harringtonine-assisted ribo-seq data [15, 16].

Since microRNAs inhibit translation of their target mRNAs, theoretically this inhibition may at least partly explain the differences between the gene expression profiles deduced from the total mRNA sequencing and from the ribo-seq profiling data, both at the level of single genes and molecular pathways.

Similar to CNR and PAS concepts for protein-coding genes and their transcripts, the corresponding values describing impact of total microRNA profiles on translation, can be calculated. Recently, a formula was published for the miRNA-based pathway activation strength calculations [17, 18]. For any distinct protein-coding gene *g* inhibited by microRNAs, a value indicating miRNA impact on its regulation, is introduced:$\log\text{CNR\_miRNA}_{g}=-\sum_{n} \text{log}\left(\text{miCNR}_{n}\right),$

where the summation is performed for all miRNA types that specifically inhibit the expression of gene *g*; miCNR*_n_* here is the case-to-normal ratio of expression levels for a particular microRNA *n*.

The miRNA-dependent effect on the activation of a pathway *p* may be evaluated as follows:

$$\text{miRNA\_PAS}_{p}=-\sum_{i} \text{ARR}_{ip}\cdot\sum_{j} \text{miII}_{ij}\cdot\text{log}\left(\text{miCNR}_{j}\right)\cdot$$

The corresponding Boolean micro-RNA involvement index (miII*_ij_*) shows whether transcript of gene $i$ is targeted by microRNA *j*. Since microRNA-induced inhibition affects translation process, theoretically the difference (*Δ* = Ribo_Seq – mRNA_Seq) between Ribo-seq and total mRNA-seq profiling results may be positively correlated with the vector driven by microRNA-induced effect on translation, at both levels: (1) at the level of individual protein-coding genes (log CNR_miRNA) and (2) at the level of molecular pathways (miRNA_PAS).

The effect of an NGS expression filter on this type of correlation is shown on Figure S2_2. The panels show how the above difference *Δ* depends on the microRNA-induced inhibition of translation. Left panels (**a**, **c** and **e**) show this dependence for the individual protein-coding genes, right panels (**b**, **d** and **f**) represent pathway-based data (PAS).

Without NGS expression filter, there is no correlation between microRNA-dependent alteration of translation and difference between ribo-seq vs total mRNA sequencing profiles, both at the level of individual genes (panel **a**) and molecular pathways (panel **b**).

Table S2_1. Performance of the NGS expression filter for the PRJEB7207 dataset [14].

| Sample ID | Sample description | Number of genes with non-zero number of reads | Sequencing depth, *K/*10^6^ reads | Proportion of genes passed filtering  (1-*F*) | Number of genes passed filtering | Minimal number of reads that passed filtering |
| --- | --- | --- | --- | --- | --- | --- |
| ERR595895 | Total mRNA-seq, myoblasts, replicate 1 | 8801 | 0.63 | 20 | 1721 | 42 |
| ERR595896 | Total mRNA-seq, myoblasts, replicate 2 | 8669 | 0.38 | 14 | 1204 | 37 |
| ERR595897 | Total mRNA-seq,  myotubes, replicate 1 | 7750 | 0.46 | 17 | 1288 | 65 |
| ERR595898 | Total mRNA-seq,  myotubes, replicate 2 | 7934 | 0.64 | 20 | 1575 | 74 |
| ERR595899 | Ribo-seq with harringtonine, myoblasts, replicate 1 | 9413 | 0.57 | 18 | 1731 | 54 |
| ERR595900 | Ribo-seq with harringtonine, myoblasts, replicate 2 | 9623 | 0.66 | 21 | 1952 | 59 |
| ERR595901 | Ribo-seq with harringtonine, myoblasts, replicate 3 | 9605 | 0.86 | 25 | 2363 | 67 |
| ERR595902 | Ribo-seq with cyclohexamide,  myoblasts, replicate 1 | 9210 | 0.36 | 13 | 1233 | 50 |
| ERR595903 | Ribo-seq with cyclohexamide,  myoblasts, replicate 2 | 9237 | 0.34 | 13 | 1190 | 49 |
| ERR595904 | Ribo-seq with cyclohexamide,  myoblasts, replicate 3 | 9050 | 0.44 | 16 | 1465 | 59 |
| ERR595905 | Ribo-seq with harringtonine, myotubes, replicate 1 | 8608 | 0.20 | 9 | 764 | 36 |
| ERR595906 | Ribo-seq with harringtonine, myotubes, replicate 2 | 8510 | 0.21 | 9 | 772 | 39 |
| ERR595907 | Ribo-seq with harringtonine, myotubes, replicate 3 | 9212 | 0.42 | 16 | 1439 | 49 |
| ERR595908 | Ribo-seq with cyclohexamide,  myotubes, replicate 1 | 9697 | 1.17 | 27 | 2654 | 79 |
| ERR595909 | Ribo-seq with cyclohexamide,  myotubes, replicate 2 | 9659 | 1.02 | 26 | 2509 | 59 |
| ERR595910 | Ribo-seq with cyclohexamide,  myotubes, replicate 3 | 10292 | 3.81 | 44 | 4573 | 114 |

When applying additional filtering to analyze only those cases that have different signs for total mRNA-seq and ribo-seq profiles (i.e., either the log-CNR/ PAS for total mRNA-seq is positive when the same value for ribo seq is negative, or vice versa), panels **e** and **f,** this dramatically increases the correlation for both levels of individual genes (panel **e**) and molecular pathways (panel **f**).

Interestingly, the pathways forming the lower cluster on this panel mostly represent various branches of the integrin-linked kinase (ILK) superpathway. The central role of the ILK signaling in the development of myotube was mentioned in many previous studies, e.g. [19, 20].


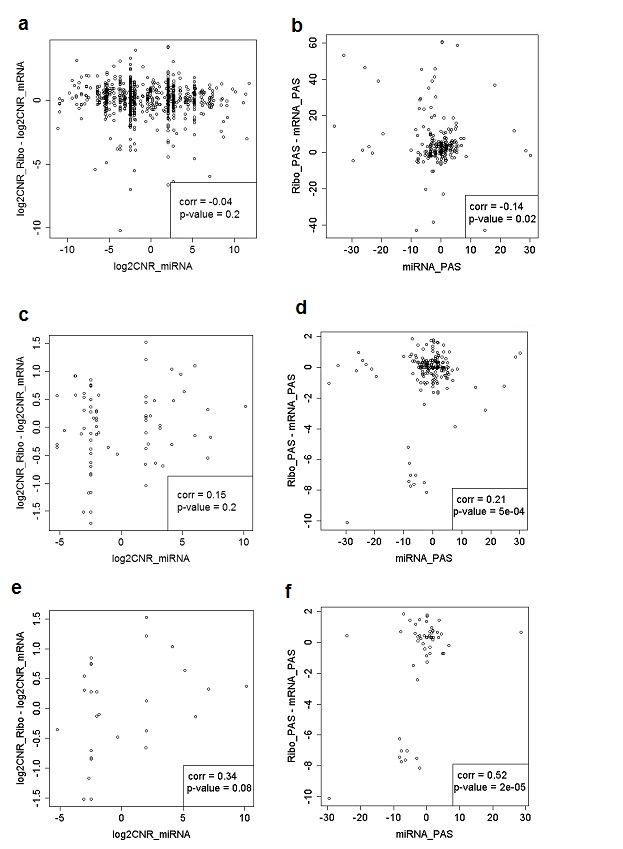


Figure S2_2. Dependence of difference (*Δ* = Ribo – mRNA) between ribo-seq and total mRNA-seq profiling results (vertical axis) upon the microRNA expression data – based predicted alteration of translation efficiencies (horizontal axis). Left panels (**a**, **c,** **e**): data analysis at the level of individual protein-coding genes (log2CNR values), right panels (**b**, **d,** **f**) represent molecular pathway (PAS) level [17, 18]. The primary expression data were obtained from [14], NEA accession number PRJEB7207 for mouse myoblasts (taken as the normal samples) and mature myotubes (taken as the case samples). Upper panels (**a,** **b**): results without NGS expression filter. Central panels (**c,** **d**): profiling after application of NGS expression filter. Lower panels (**e, f**): profiling results after application of NGS expression filter and additional selection for the entries that change their sign at the mRNA-seq and ribo-seq levels (see text).

**References**

1. GTEx Consortium. The Genotype-Tissue Expression (GTEx) project. *Nat. Genet.* 2013, 45:580–585.

2. Nakamura S, Kobayashi M, Sugino T, Kajimoto O, Matoba R, Matsubara K. Effect of exercise on gene expression profile in unfractionated peripheral blood leukocytes. *Biochem. Biophys. Res. Commun.* 2010, 391:846–851.

3. Nelson AM, Zhao W, Gilliland KL, Zaenglein AL, Liu W, Thiboutot DM. Isotretinoin temporally regulates distinct sets of genes in patient skin. *J. Invest. Dermatol.* 2009, 129:1038–1042.

4. Schoggins JW, Wilson SJ, Panis M, Murphy MY, Jones CT, Bieniasz P, Rice CM. A diverse range of gene products are effectors of the type I interferon antiviral response. *Nature* 2011, 472:481–485.

5. Haglund F, Ma R, Huss M, Sulaiman L, Lu M, Nilsson I-L, Höög A, Juhlin CC, Hartman J, Larsson C. Evidence of a functional estrogen receptor in parathyroid adenomas. *J. Clin. Endocrinol. Metab.* 2012, 97:4631–4639.

6. Ricarte-Filho JC, Li S, Garcia-Rendueles MER, Montero-Conde C, Voza F, Knauf JA, Heguy A, Viale A, Bogdanova T, Thomas GA, Mason CE, Fagin JA. Identification of kinase fusion oncogenes in post-Chernobyl radiation-induced thyroid cancers. *J. Clin. Invest.* 2013, 123:4935–4944.

7. Seo J-S, Ju YS, Lee W-C, Shin J-Y, Lee JK, Bleazard T, Lee J, Jung YJ, Kim J-O, Shin J-Y, Yu S-B, Kim J, Lee E-R, Kang C-H, Park I-K, Rhee H, Lee S-H, Kim J-I, Kang J-H, Kim YT. The transcriptional landscape and mutational profile of lung adenocarcinoma. *Genome Res.* 2012, 22:2109–2119.

8. Morrison TB, Weis JJ, Wittwer CT. Quantification of low-copy transcripts by continuous SYBR Green I monitoring during amplification. *BioTechniques* 1998, 24:954–958, 960, 962.

9. Wong ML, Medrano JF. Real-time PCR for mRNA quantitation. *BioTechniques* 2005, 39:75–85.

10. Jones GM, Busby E, Garson JA, Grant PR, Nastouli E, Devonshire AS, Whale AS. Digital PCR dynamic range is approaching that of real-time quantitative PCR. *Biomol. Detect. Quantif.* 2016, 10:31–33.

11. Xu J, Gong B, Wu L, Thakkar S, Hong H, Tong W. Comprehensive Assessments of RNA-seq by the SEQC Consortium: FDA-Led Efforts Advance Precision Medicine. *Pharmaceutics* 2016, 8.

12. Ramos A, Santos C, Barbena E, Mateiu L, Alvarez L, Nogués R, Aluja MP. Validated primer set that prevents nuclear DNA sequences of mitochondrial origin co-amplification: a revision based on the New Human Genome Reference Sequence (GRCh37). *Electrophoresis* 2011, 32:782–783.

13. Schneider VA, Graves-Lindsay T, Howe K, Bouk N, Chen H-C, Kitts PA, Murphy TD, Pruitt KD, Thibaud-Nissen F, Albracht D, Fulton RS, Kremitzki M, Magrini V, Markovic C, McGrath S, Steinberg KM, Auger K, Chow W, Collins J, Harden G, Hubbard T, Pelan S, Simpson JT, Threadgold G, Torrance J, Wood JM, Clarke L, Koren S, Boitano M, Peluso P, Li H, Chin C-S, Phillippy AM, Durbin R, Wilson RK, Flicek P, Eichler EE, Church DM. Evaluation of GRCh38 and de novo haploid genome assemblies demonstrates the enduring quality of the reference assembly. *Genome Res.* 2017, 27:849–864.

14. de Klerk E, Fokkema IFAC, Thiadens KAMH, Goeman JJ, Palmblad M, den Dunnen JT, von Lindern M, Hoen PAC. Assessing the translational landscape of myogenic differentiation by ribosome profiling. *Nucleic Acids Res.* 2015, 43:4408–4428.

15. Ingolia NT, Lareau LF, Weissman JS. Ribosome profiling of mouse embryonic stem cells reveals the complexity and dynamics of mammalian proteomes. *Cell* 2011, 147:789–802.

16. Volkova OA, Kondrakhin YV, Yevshin IS, Valeev TF, Sharipov RN. Assessment of translational importance of mammalian mRNA sequence features based on Ribo-Seq and mRNA-Seq data. *J. Bioinform. Comput. Biol.* 2016, 14:1641006.

17. Artcibasova AV, Korzinkin MB, Sorokin MI, Shegay PV, Zhavoronkov AA, Gaifullin N, Alekseev BY, Vorobyev NV, Kuzmin DV, Kaprin АD, Borisov NM, Buzdin AA. MiRImpact, a new bioinformatic method using complete microRNA expression profiles to assess their overall influence on the activity of intracellular molecular pathways. *Cell Cycle Georget. Tex* 2016, 15:689–698.

18. Buzdin AA, Artcibasova AV, Fedorova NF, Suntsova MV, Garazha AV, Sorokin MI, Allina D, Shalatonin M, Borisov NM, Zhavoronkov AA, Kovalchuk I, Kovalchuk O, Kushch AA. Early stage of cytomegalovirus infection suppresses host microRNA expression regulation in human fibroblasts. *Cell Cycle Georget. Tex* 2016, 15:3378–3389.

19. Grzelkowska-Kowalczyk K, Tokarska J, Grabiec K, Gajewska M, Milewska M, Błaszczyk M. Tumor necrosis factor-α alters integrins and metalloprotease ADAM12 levels and signaling in differentiating myoblasts. *Pol. J. Vet. Sci.* 2016, 19:253–259.

20. Wu H, Ren Y, Pan W, Dong Z, Cang M, Liu D. The mammalian target of rapamycin signaling pathway regulates myocyte enhancer factor-2C phosphorylation levels through integrin-linked kinase in goat skeletal muscle satellite cells. *Cell Biol. Int.* 2015, 39:1264–1273.
