# Supplementary figures and images for: Shambhala: a platform-agnostic data harmonizer for gene expression data
Source: BMC Bioinformatics. 2019 Feb 6;20:66. doi: 10.1186/s12859-019-2641-8 (PMC6366102; doi:10.1186/s12859-019-2641-8)

## Slide 1
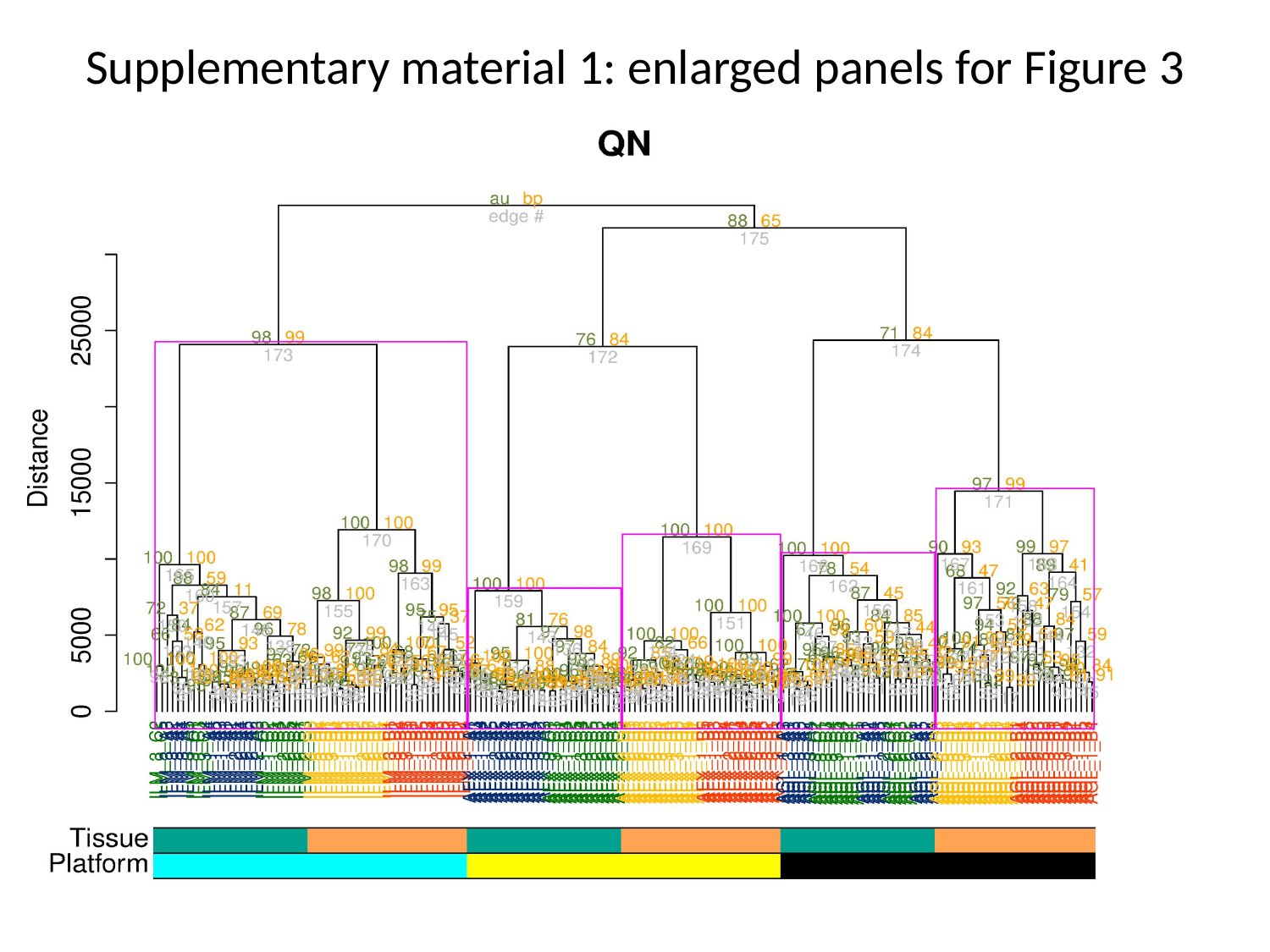

Supplementary material 1: enlarged panels for Figure 3

## Slide 2
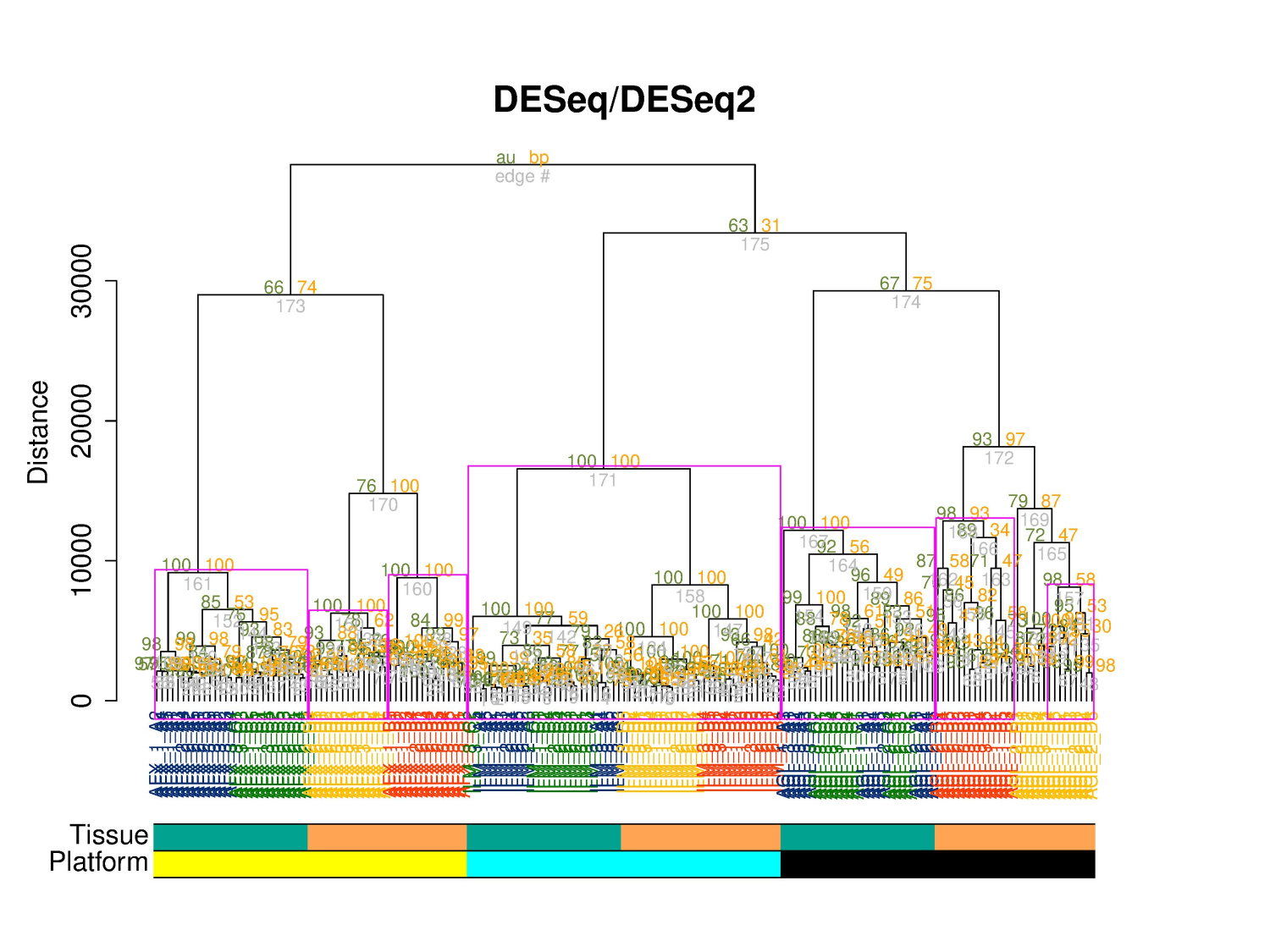

## Slide 3
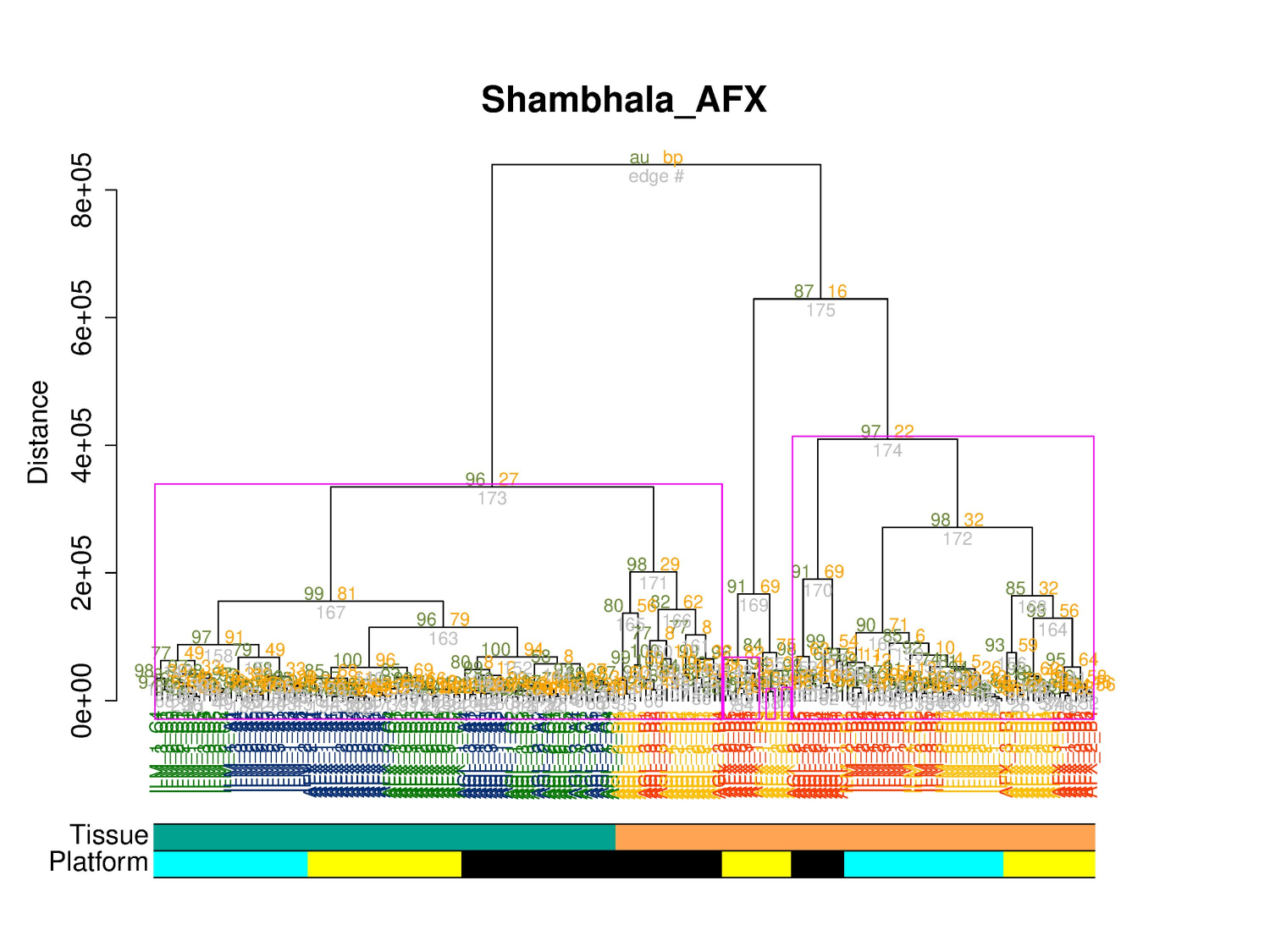

## Slide 4
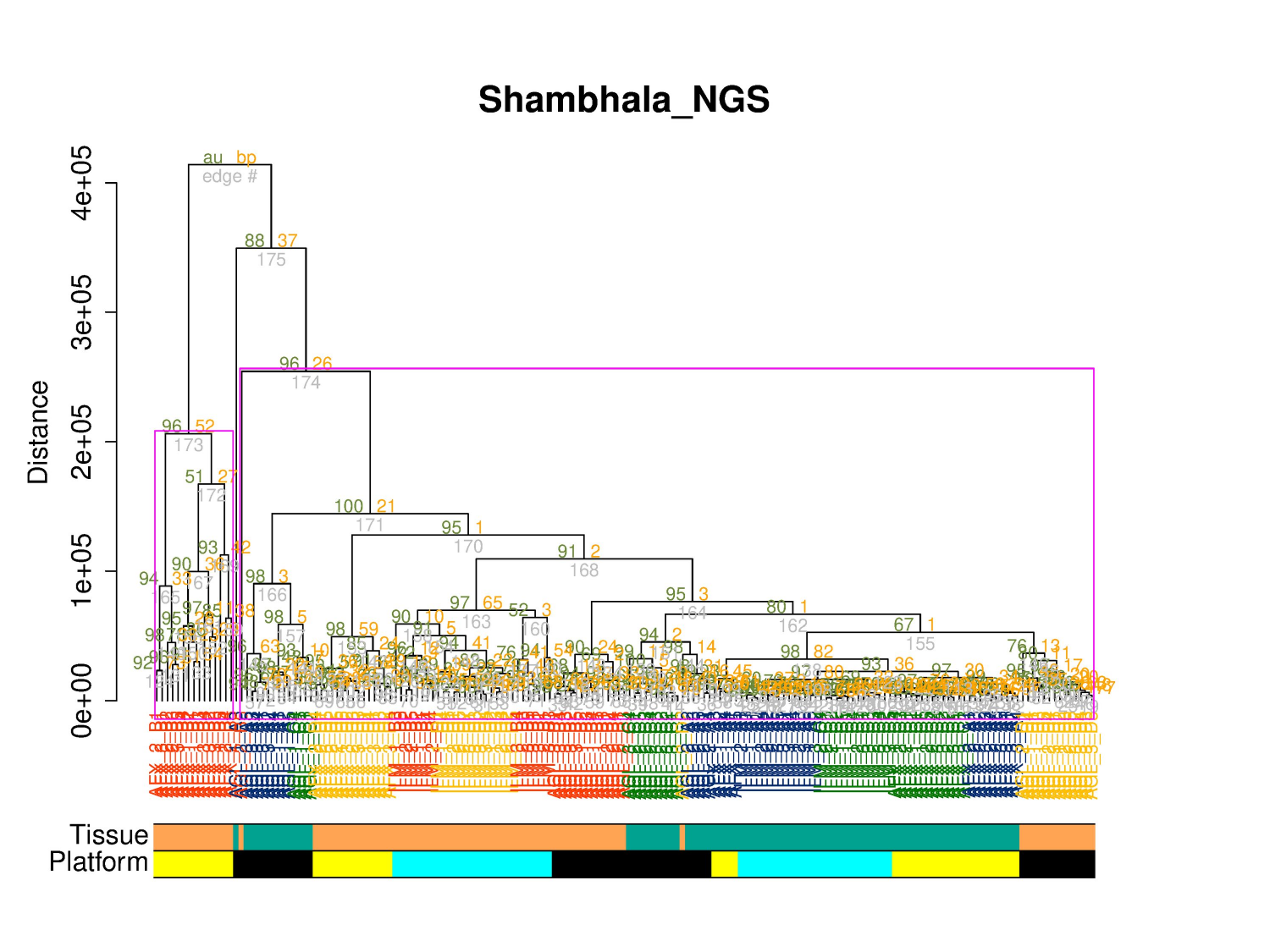

## Slide 5
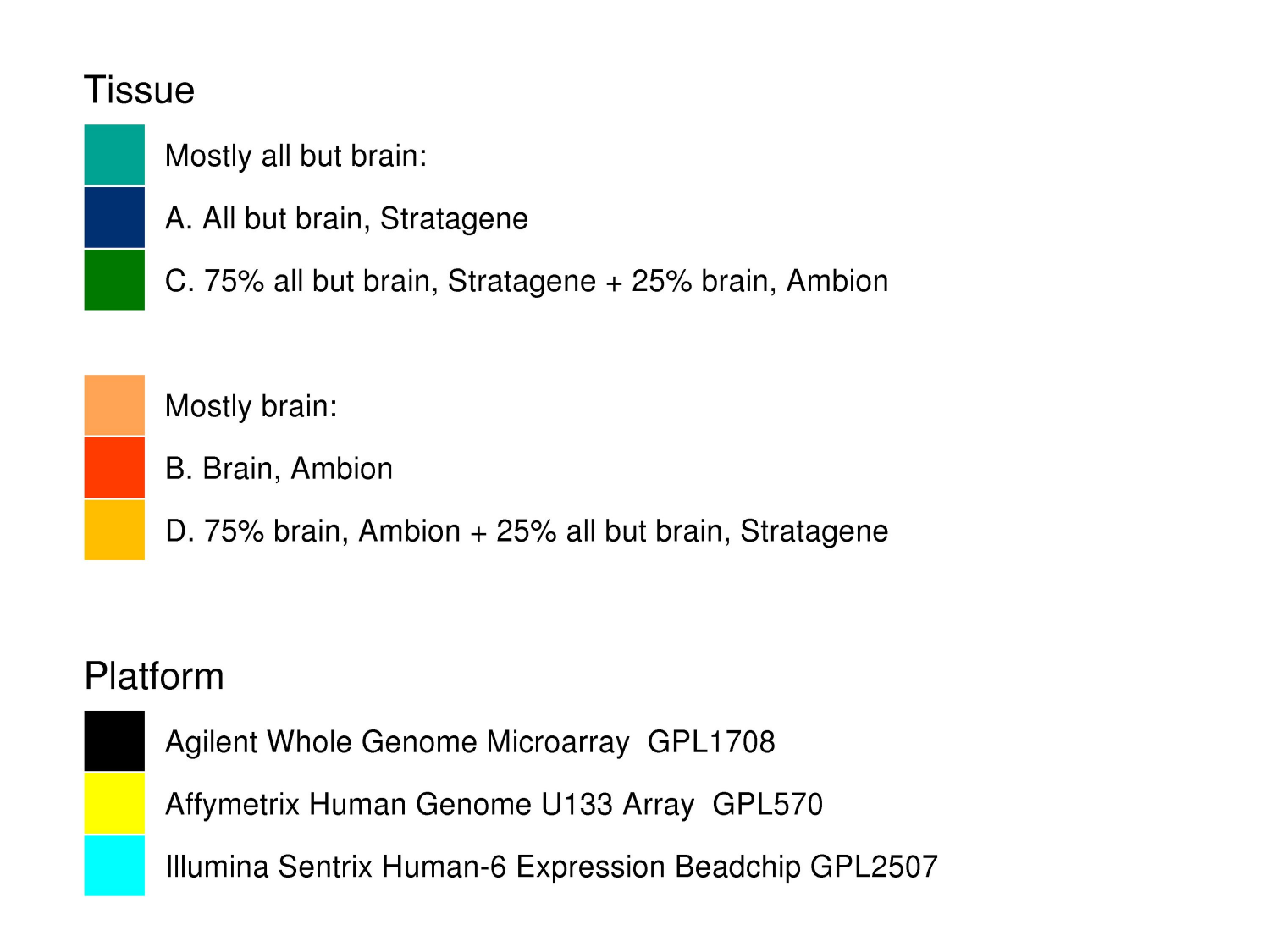

Supplement: Supplementary file 5 — A detailed view of hierarchical clustering for gene expression levels for MAQC project data after application different harmonization methods. (PPTX 857 kb) [file 12859_2019_2641_MOESM5_ESM.pptx]

## Slide 1
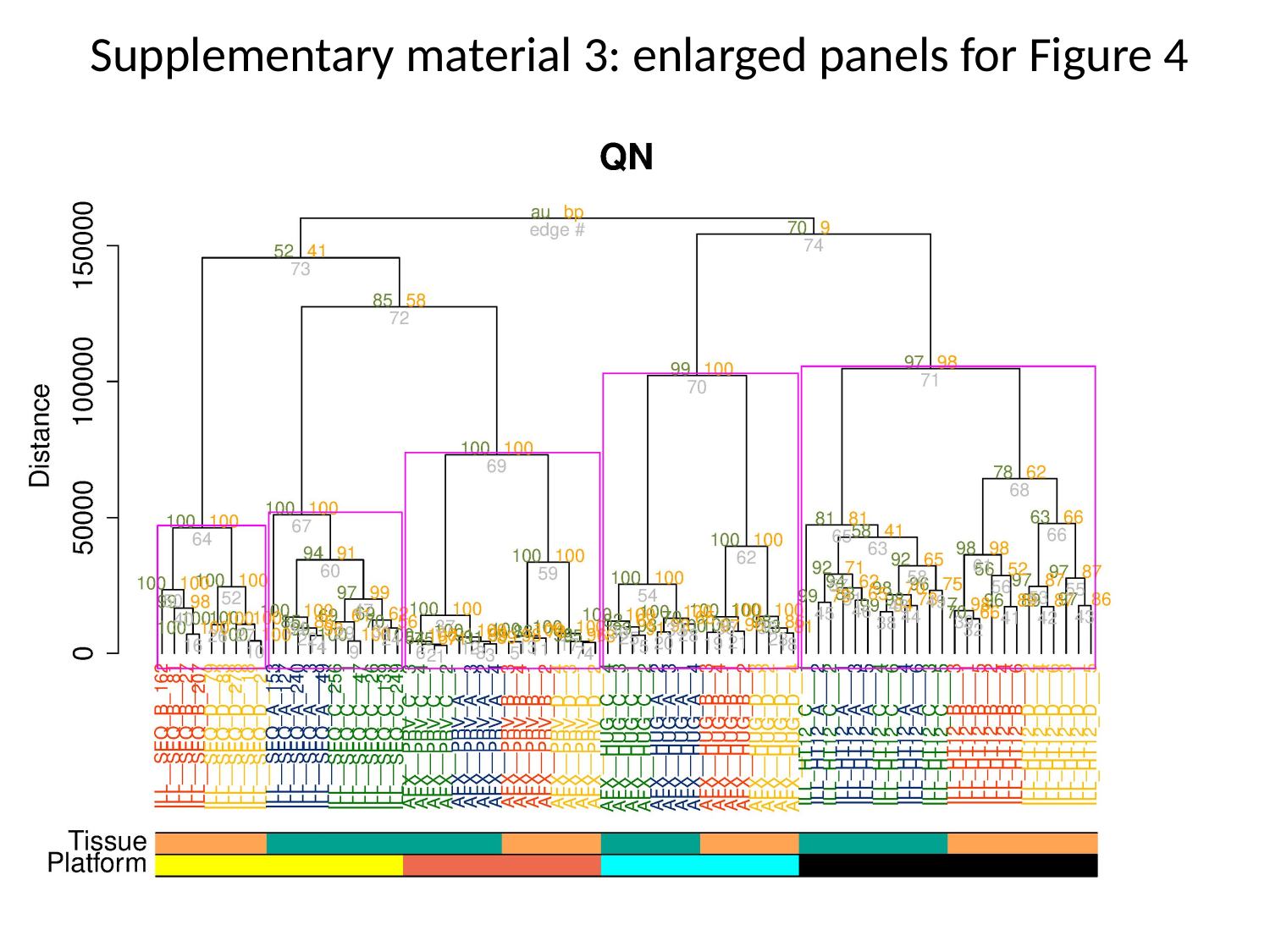

Supplementary material 3: enlarged panels for Figure 4

## Slide 2
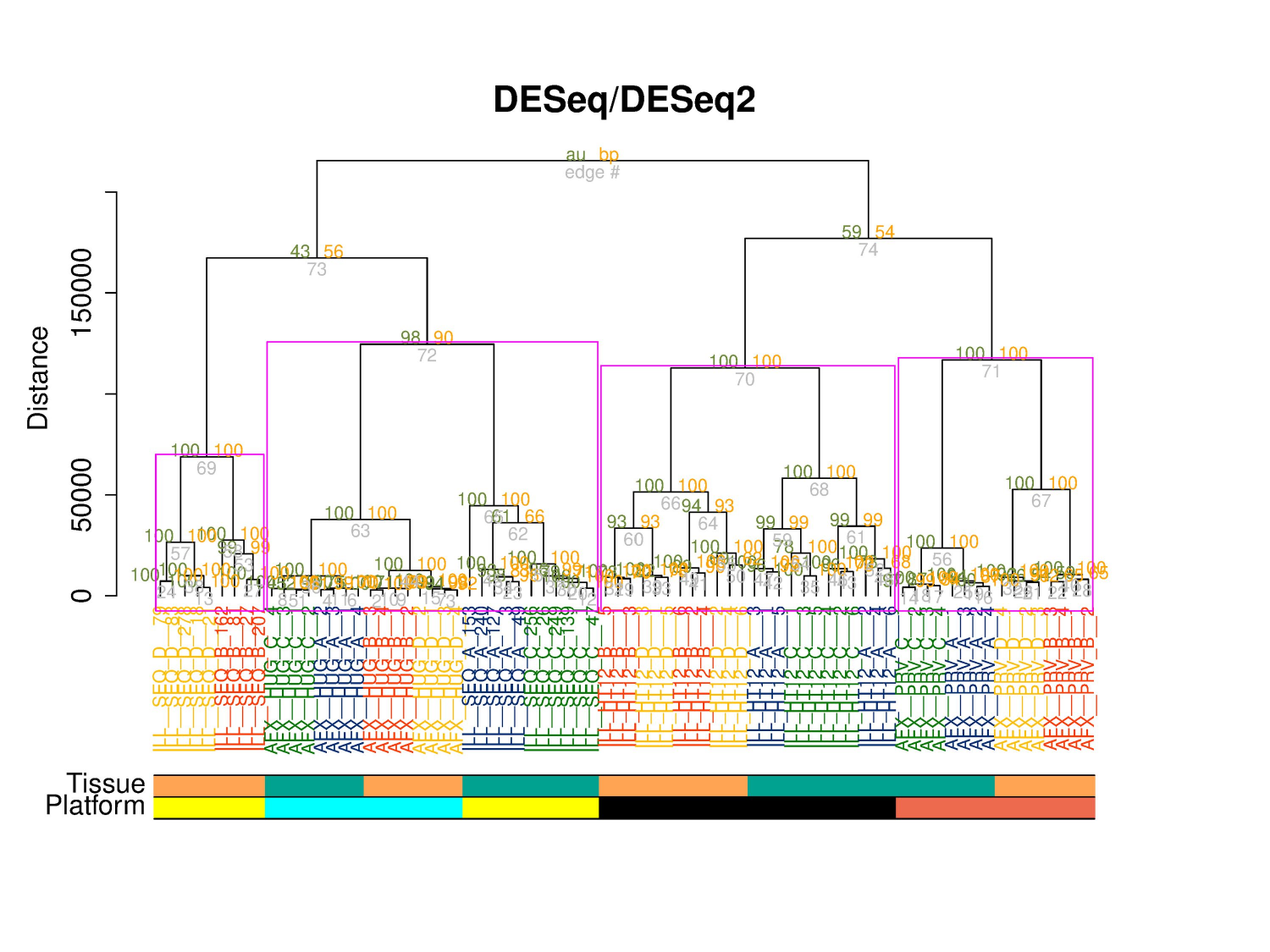

## Slide 3
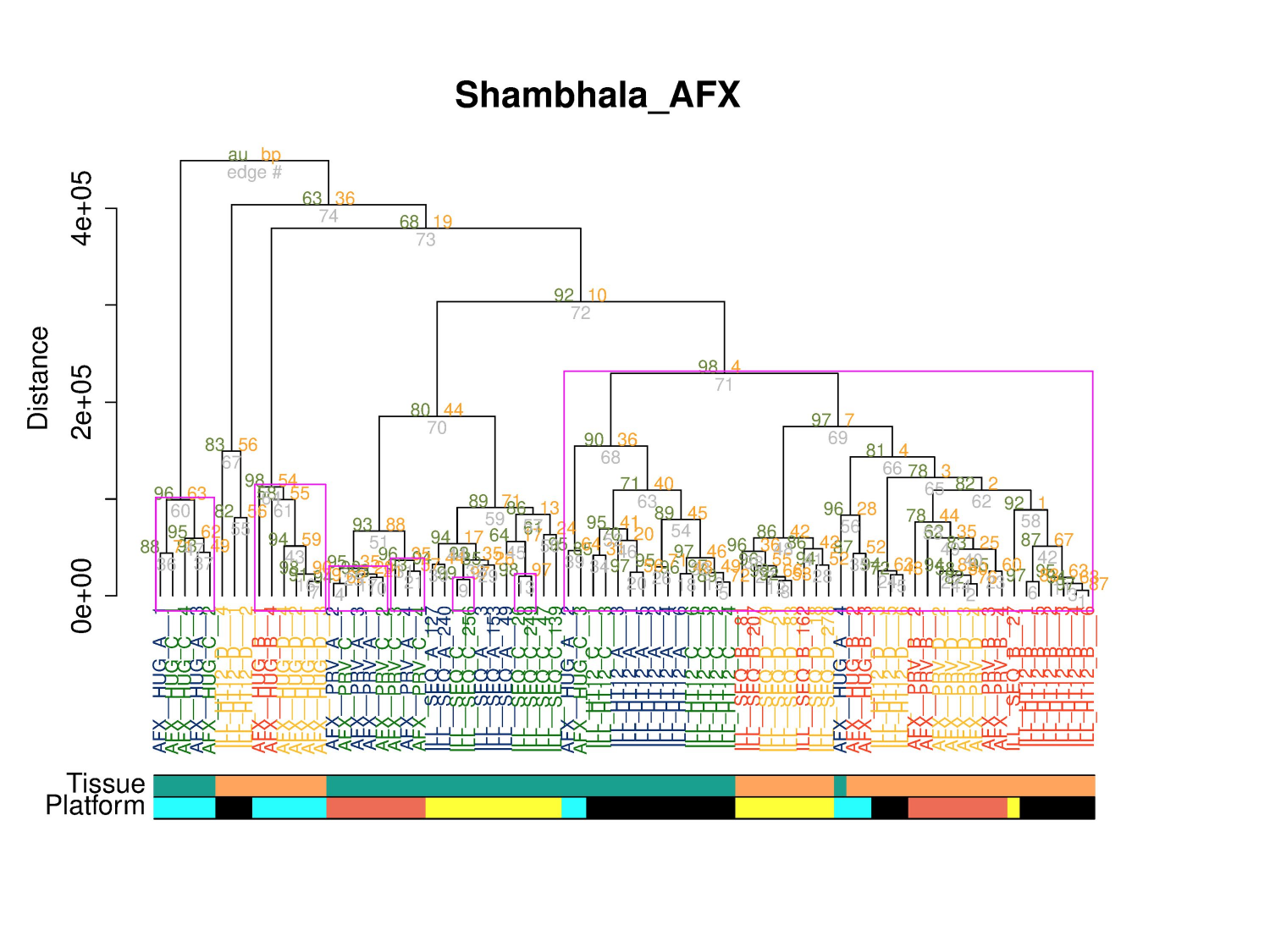

## Slide 4
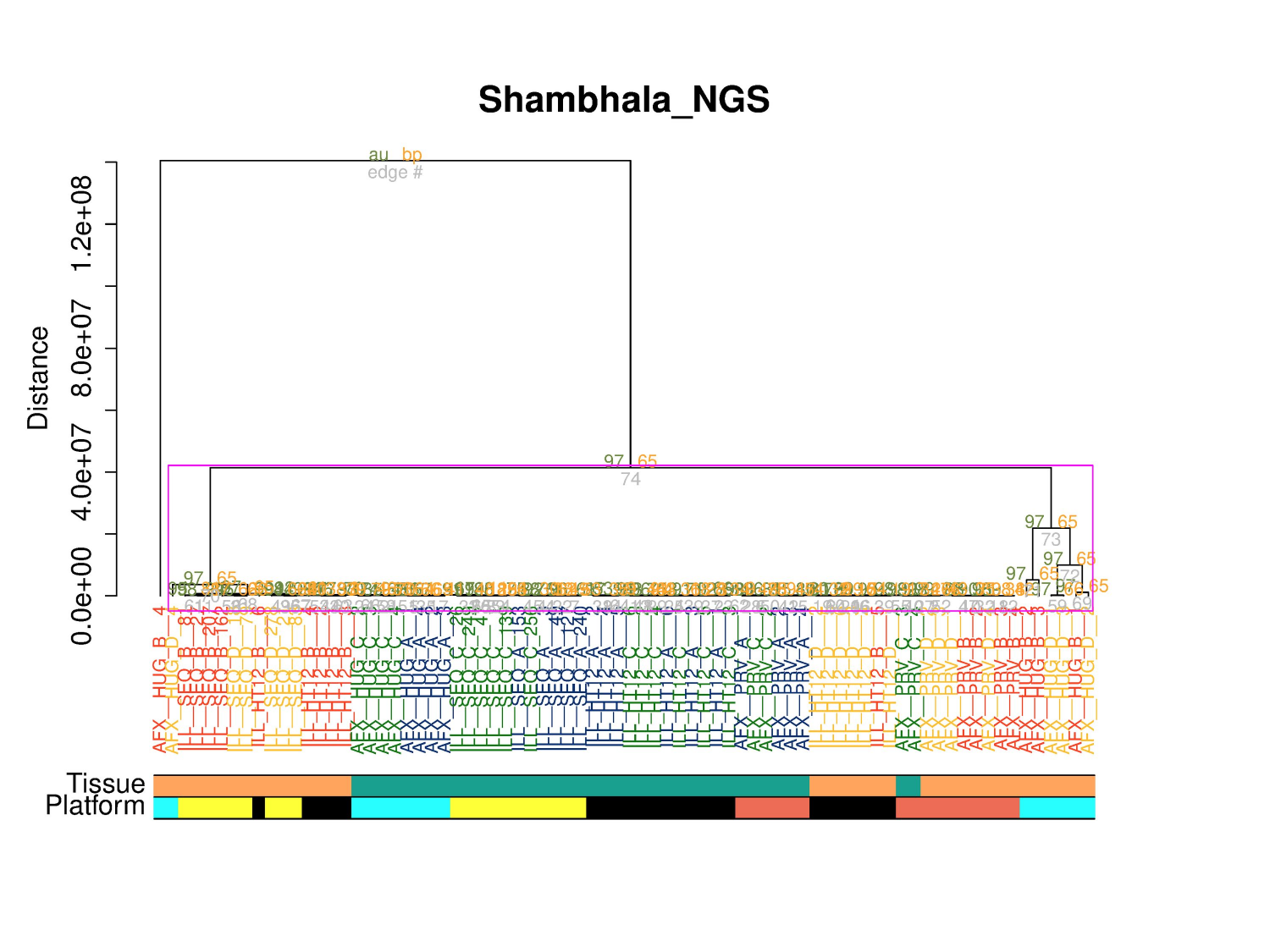

## Slide 5
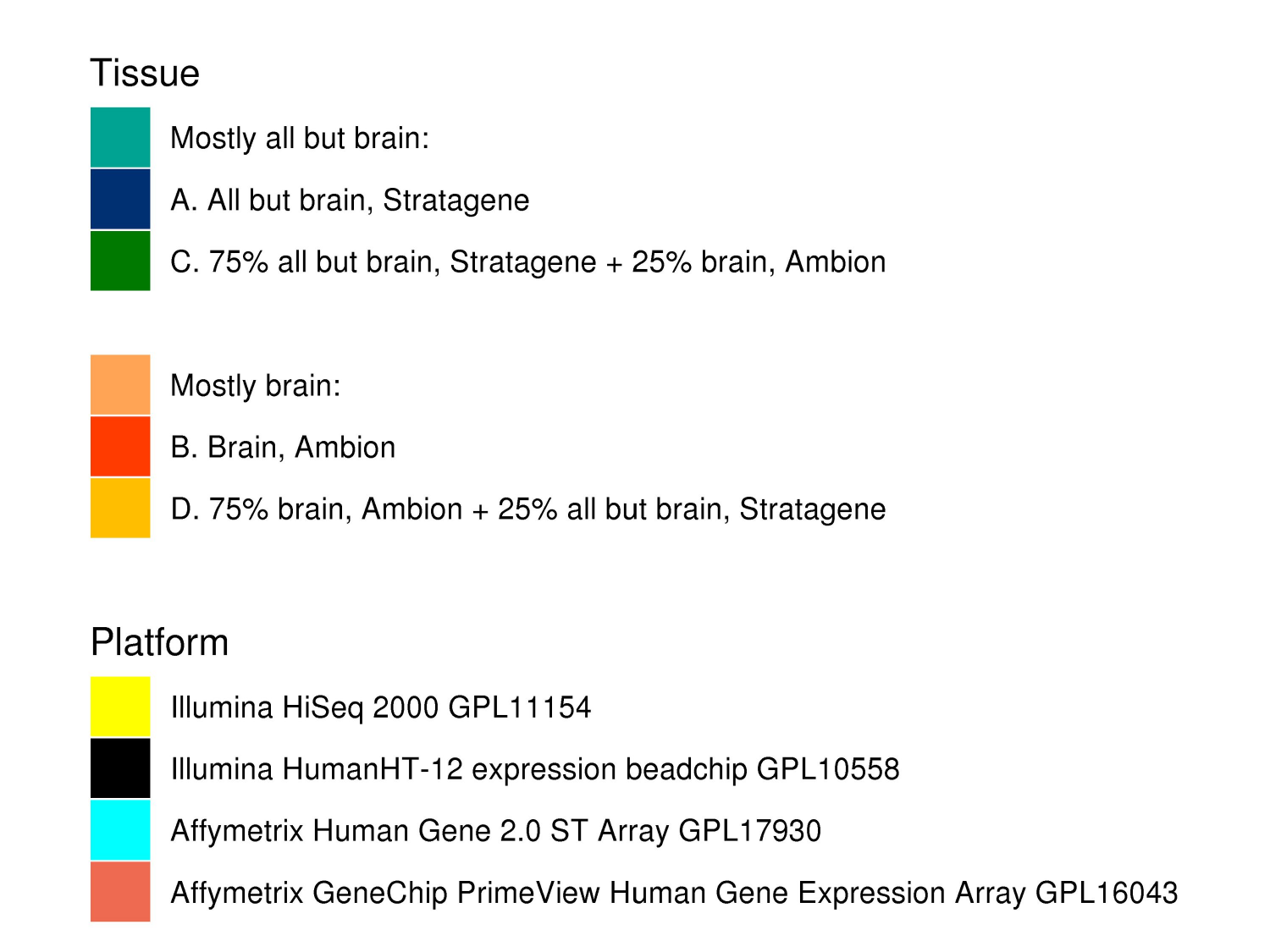

Supplement: Supplementary file 6 — A detailed view of hierarchical clustering for gene expression levels for SEQC project data after application different harmonization methods. (PPTX 647 kb) [file 12859_2019_2641_MOESM6_ESM.pptx]
